# Postpartum pulmonary micronodules and thyroid cystic nodules in a COVID-19 vaccinated patient: A CARE-compliant case report

**DOI:** 10.1097/MD.0000000000045557

**Published:** 2025-10-24

**Authors:** Yinling Chen, Jie Huang, Xinyan Chen, Ruitao Zheng

**Affiliations:** a School of Medicine, Hangzhou City University, Hangzhou, China; b Anji People’s Hospital, Affiliated Anji Hospital, School of Medicine, Hangzhou City University, Huzhou, China.

**Keywords:** COVID-19 vaccination, gestational stress, lifestyle modifications, pulmonary micronodules, thyroid cystic nodules

## Abstract

**Rationale:**

This study presents the first documented case suggesting a potential association between prepregnancy administration of 4 doses of mRNA corona virus disease-19 vaccine and the development of postpartum multi-organ nodules, including pulmonary micronodules and thyroid cysts. The purpose of this report is to delineate a clinical scenario involving 3 interrelated factors: vaccine-induced sustained immune activation, inadequately managed moderate anxiety during pregnancy, and significant lifestyle alterations such as a gluten- and dairy-free diet coupled with sleep deprivation. The significance of this case lies in its novel exposure profile and undocumented long-term implications, offering critical insights that may inform future reproductive health guidance and risk counseling.

**Patient concerns:**

A 34-year-old women who presented with incidental findings of asymptomatic bilateral diffuse pulmonary micronodules and thyroid cystic nodules (TI-RADS 1) during routine 6-month postpartum imaging.

**Diagnoses:**

Chest computed tomography and thyroid ultrasound confirmed multiple pulmonary micronodules coexisting with benign thyroid cystic nodules. Serial investigations ruled out metastasis, granulomatous disease, and classic autoimmune disorders.

**Interventions:**

A diagnostic monitoring strategy was implemented, including serial pulmonary computed tomography (6-month follow-ups) and thyroid ultrasound surveillance (12-month follow-ups), complemented by lifestyle rebalancing (gradual exercise reintroduction/sleep optimization) and psychological counseling for health-related anxiety. Pharmacological intervention was withheld to observe the natural evolution of nodule regression and immune homeostasis restoration.

**Outcomes:**

During the ongoing follow-up period, there were no other improvements or deterioration developments.

**Lessons:**

This case suggests prepregnancy vaccine immune imprinting may become activated under gestational stress, potentially triggering subclinical inflammation via the hypothalamic-pituitary-adrenal axis-gut microbiota-cytokine network. Consequently, we propose the “V-SLAM” clinical framework (vaccine history/stress biomarkers/lifestyle factors/autoimmunity screening/multidisciplinary consultation), emphasizing the integration of vaccination records and psychological resilience assessment into preconception counseling, with cross-pregnancy immune-metabolic monitoring for high-risk women.

## 1. Introduction

The postpartum period represents a critical window for detecting subclinical physiological changes. Recent clinical observations have noted increased incidence of benign pulmonary and thyroid nodules in postpartum women, with potential links to 3 emerging factors: corona virus disease (COVID)-19 vaccination effects, pregnancy-associated psychological stress, and gestational lifestyle modifications. While current literature has examined these factors independently, their combined effects remain poorly understood. This case is particularly noteworthy because of the extended interval between vaccination and nodule detection, spanning preconception through postpartum periods. We present detailed clinical, laboratory, and imaging data with comprehensive analysis of potential mechanistic interactions.

## 2. Case report

A 34-year-old primigravida (body mass index 21.9 kg/m²) presented for routine postpartum care 6 months after uncomplicated vaginal delivery. She reported no respiratory symptoms or neck discomfort.

### 2.1. Medical history

COVID-19 vaccination: 4 doses mRNA vaccine, 12 months preconception; no SARS-CoV-2 infection history; and mild intermittent asthma (during pregnancy).

### 2.2. Pregnancy course

General anxiety disorder-7 score: 10 (moderate anxiety); Pittsburgh sleep quality index score: 8 (poor sleep quality).

### 2.3. Dietary changes

Increased soy and dairy intake.

### 2.4. Physical activity

Reduced from 8000 to 2000 steps/day.

### 2.5. Imaging findings

**Figure 1.**
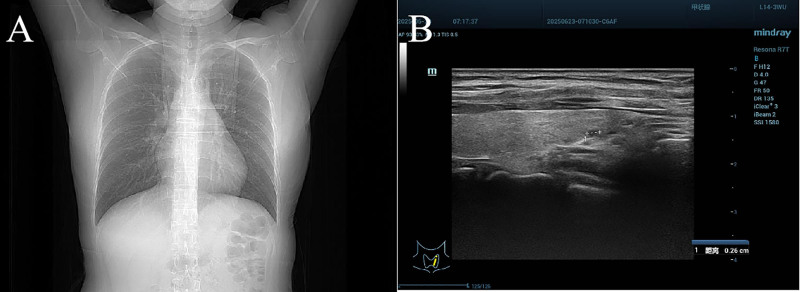
Representative imaging of chest CT and thyroid ultrasound. (A) Representative imaging for pulmonary micronodules by chest CT; (B) representative imaging for thyroid cystic nodule via thyroid ultrasound. CT = computed tomography.

Chest computed tomography (CT): 3 to 5 randomly distributed micronodules (1–3 mm), no lymphadenopathy (Fig. 1A).

### 2.6. Thyroid ultrasound

Two cystic nodules (largest 0.26 cm in left lobe); TI-RADS 1 classification; normal vascularity (Fig. 1B).

### 2.7. Laboratory results

Thyroid stimulating hormone: 1.48 mIU/L (normal); free triiodothyronine 3: 2.85 ng/L (normal); FT4: 7.38 ng/L (normal). Inflammatory markers: C-reactive protein 1.4 mg/L (normal).

## 3. Clinical outcomes

A 34-year-old primigravida, with a notable history of 4 doses of mRNA COVID-19 vaccine administered, was found to have developed multi-organ nodules during her postpartum evaluation. Imaging studies revealed randomly distributed pulmonary micronodules (1–3 mm) on chest CT and 2 cystic thyroid nodules (largest 0.26 cm), despite the patient being completely asymptomatic. This clinical presentation occurred in the context of moderate anxiety, poor sleep quality during pregnancy, and significant lifestyle modifications including markedly reduced physical activity and dietary changes. All thyroid function tests and inflammatory markers remained within normal limits. This case represents the first documented potential association between prepregnancy mRNA COVID-19 vaccination and the development of postpartum multi-organ nodules, highlighting a novel clinical trajectory that warrants further investigation.

## 4. Discussion

### 4.1. Prepregnancy vaccination and delayed immuneeffects

Emerging evidence suggests that the 18-month interval between COVID-19 mRNA vaccination and subsequent detection of pulmonary and thyroid nodules may reflect prolonged immune activation pathways that were potentially amplified by subsequent pregnancy.^[1]^ Current research indicates that mRNA vaccines can induce sustained germinal center reactions persisting up to 6 months postvaccination, along with delayed resolution of vaccine-related inflammation, which may have been further modulated by pregnancy-induced immune adaptations.^[2]^ The underlying mechanisms for this prolonged immune response likely involve several interconnected biological processes: persistent spike protein antigen presentation may maintain low-grade immune stimulation^[3]^; pregnancy-related hormonal changes could have amplified preexisting vaccine-induced immune activation through epigenetic modifications in immune cells^[4]^; and the development of cross-reactive autoantibodies might have contributed to inflammatory microenvironments in susceptible tissues.^[5]^ This extended immune activation timeline is particularly noteworthy as it bridges the preconception vaccination period through gestation and into the postpartum phase, suggesting that vaccine-related immune modifications may create a biological “priming” effect that interacts with subsequent physiological stressors during pregnancy.^[6]^ The case highlights the complex temporal dynamics of vaccine responses in women of reproductive age, where prepregnancy immunological changes may potentially influence tissue responses to the unique immunologic challenges of gestation and postpartum recovery.

### 4.2. Gestational stress and endocrine disruption

The patient’s moderate anxiety during pregnancy (general anxiety disorder-7 score of 10) likely contributed to nodule formation through 3 interconnected stress-mediated pathways. First, chronic stress activated the hypothalamic-pituitary-adrenal (HPA) axis, leading to sustained cortisol elevation that significantly impaired immune homeostasis.^[7]^ Second, this triggered a pro-inflammatory cascade characterized by elevated IL-6 and TNF-α production, maintaining systemic low-grade inflammation throughout gestation.^[8]^ Third, stress-induced alterations in deiodinase activity modified local thyroid hormone metabolism, potentially predisposing to cystic changes.^[9]^ These pathways collectively created a permissive environment where neuroendocrine dysregulation (cortisol increased), chronic inflammation (IL-6/TNF-α increased), and thyroid dysfunction converged to promote nodule development. The duration of stress exposure (entire pregnancy) was particularly significant, as prolonged HPA axis activation may have caused cumulative tissue-level effects that manifested postpartum.

### 4.3. Lifestyle factors and nodule development

The patient demonstrated 3 clinically significant lifestyle modifications during pregnancy that may have contributed to her postpartum findings. First, notable dietary changes included increased soy consumption (4–5 servings/wk) containing phytoestrogens, regular dairy intake, and use of high-iodine prenatal vitamins (150 μg/d) – factors known to influence thyroid morphology.^[10]^ Second, physical activity markedly declined by 75% from prepregnancy levels, reducing important cardioprotective and anti-inflammatory benefits.^[11]^ Third, sleep disturbances developed, characterized by reduced sleep efficiency and elevated serum biomarkers.^[12]^ These modifications collectively created a pro-oxidative, pro-inflammatory state that may have potentiated tissue changes.

### 4.4. Management and follow-up

The patient’s management plan included: Diagnostic follow-up with 6-month CT and thyroid ultrasound, plus negative autoimmune panel; Therapeutic interventions featuring stress reduction counseling, dietary modification, and gradual exercise reintroduction; and Long-term monitoring with annual thyroid ultrasounds, pulmonary function tests, and inflammatory marker tracking. This comprehensive approach balanced surveillance with lifestyle optimization.

### 4.5. Limitations

As a single case report, this study cannot establish causality and is subject to inherent recall bias and unmeasured confounding factors. The complex interplay of multiple exposures also makes it difficult to attribute the observed outcomes to any single element. Generalizability is limited, and the findings should be interpreted as hypothesis-generating for further investigations.

## 5. Conclusions

This case highlights the complex interplay between prepregnancy COVID-19 vaccination (inducing prolonged immune activation), gestational stress (causing HPA axis dysregulation), and lifestyle factors (diet/exercise/sleep changes) in postpartum nodule development. We propose a novel 3-dimensional framework (vaccine-stress-lifestyle) emphasizing: preconception vaccination may prime immune responses, pregnancy represents a critical window for stress-induced endocrine disruption, and lifestyle modifications create a permissive pro-inflammatory microenvironment. This integrative approach helps differentiate physiological adaptations from pathological changes requiring intervention, with particular attention to the temporal sequence of events from preconception through postpartum periods.

## Acknowledgments

The authors gratefully acknowledge all the participants for their support in this research letter. We would like to thank Xiaoxi Zhang for English proofing.

## Author contributions

**Conceptualization:** Yinling Chen.

**Data curation:** Jie Huang.

**Writing – original draft:** Yinling Chen.

**Writing – review & editing:** Jie Huang, Xinyan Chen, Ruitao Zheng.
